# Why continuous discussion can promote the consensus of opinions?

**DOI:** 10.1186/s40649-016-0035-x

**Published:** 2016-11-21

**Authors:** Zhenpeng Li, Xijin Tang, Benhui Chen, Jian Yang, Peng Su

**Affiliations:** 1 Department of Applied Statistics, Dali University, Dali, 671003 China; 2 Academy of Mathematics and Systems Sciences, Chinese Academy of Sciences, Beijing, 100190 China

**Keywords:** Social influence network theory, Random graph, Opinions dynamics

## Abstract

Why group opinions tend to be converged through continued communication, discussion and interactions? Under the framework of the social influence network model, we rigorously prove that the group consensus is almost surely within finite steps. This is a quite certain result, and reflects the real-world common phenomenon. In addition, we give a convergence time lower bound. Although our explanations are purely based on mathematic deduction, it shows that the latent social influence structure is the key factor for the persistence of disagreement and formation of opinions convergence or consensus in the real world social system.

## Background

Many social phenomena are embedded within networks of interdependencies, i.e., social networks or social structures. The social structure are constructed by social ties (or social connections) as suggested by Granovetter. Different types of connections might play different roles for the function of network. For example, Granovetter argued that weak ties (individuals are loosely connected in the network) were the necessary condition for spreading to occur across subnetwork within a social system [1]. Burt suggested that structural equivalence is the factor for the adoption of new ideas [2]. In the field of complex networks, researchers focus both the structure and function of social networks, i.e., networks dynamical processes taking place on networks, such as the transmission of disease over human contact and rumors diffusion through internet [3].

Inspired by these researches, instead of discussing spreading or innovation adoption in network, the present research ask how do social networks (different social structures) influence opinions dynamics? i.e., the influence of social network structure on opinions dynamics, we will investigate under what conditions opinions in a group can reach consensus and average consensus.

Here we refer to the network is the pattern of friendship, advice, communication, support or is the form of bargaining, debating and compromising. In the structured social context, individual determines his/her opinions, in accordance with the constraints and possibilities imposed by other’s in the network. In other words, individuals are assumed to be responsive to the contextual cues provided by the opinions and behavior of significant others. Through advice, communication, support or bargaining, debating and compromising, consecutively, actors thus establish their own behavior, by appropriately taking into account the opinions and behaviors displayed by their significant others.

In the end, the aggregation of local individual’s opinions adjustment contribute to the global group opinions patterns—*polarization or consensus*. In social psychology, during the past decades, group polarization phenomenon has been intensively studied [4–9].

The importance of group polarization is significant as it helps to explain group behavior in a variety of real-life situations. Examples of these situations include public voting, terrorism, and violence. In our former studies, we investigated the group polarization based on Hopfield attractor model, and revealed a very interesting connection between global patterns and local structure balance [10]. Next, we will concentrate on the phenomenon of group opinions convergence and consensus.

Mathematical models are used to describe consensus include DeGroot’s classic model [11], Friedkin and Johnsen [12] and Friedkin’s extended version [13]. From social psychological point of view, this line of research began with French’s formal theory of social power [14], a simple model of collective opinion formation in a network of interpersonal influencing social group. As a step forward, Friedkin presented the social influence network theory based on Latane’s social influence theory [15], which considered both cognitive and structural aspects, and focused on the contributions of networks of interpersonal influence to the formation of interpersonal agreements and group consensus.

Over the past few years, models of the convergence of opinion or consensus problem in social systems have been the subjects of a considerable amount of recent attention in the fields such as motion coordination of autonomous agents [16, 17], distributed computation in control theory [16, 18, 19], randomized consensus algorithms [20, 21], and sensor networks about data fusion problems [22–26].

Due to the unpredictability of the environment where the communication between agents occurs, and the random characteristics of influences or interactions among agents in systems (man made or social systems), most of the growing interests in consensus problems (both algorithms and practical applications) are based on probabilistic settings [20].

Recently, the study of opinion dynamics has started to attract the attention of the control community, who with the bulk of motivation have developed about methods to approximate and stabilize consensus, synchronization, and other coherent states. However, comparing with many man-made or engineering systems, social systems do not typically exhibit a consensus of opinions, but rather a persistence of disagreement, i.e., polarization patterns. The ubiquitous group polarization phenomena can be observed from political election to carbon dioxide emissions debate [27]. In a social system, the difficulty in arriving at a collective consensus state roots in the fact that the process of opinion formation can rarely be reduced to accepting or rejecting the consensus of others, as exemplified by Arrow’s dilemma of social choice [28]. On the contrary, in most cases individuals construct their options in a complex interpersonal environment or with their prior identities (e.g. prior beliefs, prejudices and social identities etc.), their views are often in a state of disagreement or not easily changed, due to opinion-dependent limitations in the network connectivity and obstinacy of the agents as pointed in Ref. [29]. This phenomenon shows the complexities of social control in social economic systems.^1^

Consensus as one of the important and regular group opinions dynamic pattern is generally observed in a relative smaller group discussion and barging process. Friedkin and Johnsen’s social influence network theory emphasizes that the interpersonal influence social structure (or social influence matrix) is the underling precondition for the group consensus or opinion convergence. In that model, the initial social influence structure of group of actors is assumed to be fixed during the entire process of opinion formation. However, with the evolution of time stamp, considering both stubborn and susceptible effects, the interpersonal influence structure can be regarded as a dynamic recursive process. For this reason, the interpersonal influence structure in their model is also dynamic, as described in "Problem formulation and terminology" section.

In this paper, our aim is to investigate the precondition for consensus formation in a social group based on Friedkin’s model. From interpersonal network structure point of view, our investigation presents the conditions for the formation of group opinions convergence and consensus. We investigate the opinions convergence phenomenon over a group of *N* individuals with a random walk social influence structure, and for any given initial opinions distribution, i.e., the opinions evolution problem with a (time-variant) linear dynamic model driven by random matrices. Our analytic proof provides strict mathematic explanations for the deterministic characterization of the ergodicity, which can be used for studying the consensus over random graphs and the formation of opinion parties. The proof procedures are self-contained and based on ergodic theorem of Markov chain and eigenvalues of random graph, as introduced in Ref. [30].

The rest of the paper arranged as follows. In "Problem formulation and terminology", we will briefly introduce social influence network theory and its mathematical framework. "Random walk on weighted graph"' section present the conditions for a group opinions consensus based on random walk on weighted graph. "The convergence of opinions profile on random graph" section prove that the convergence of group opinions over general weighted and undirected random graph are almost surely. "Numeric simulation" section test the theoretical conclusion by numeric simulation methods. "Conclusions" section is our concluding remarks.

## Problem formulation and terminology

Social influence network theory presents a mathematical formalization of the social process of opinions changes that unfold in a social network of interpersonal influences. The spread of influence among individuals in a social network can be naturally modeled under a probabilistic framework. Here, we briefly describe the classical Friedkin and Johnsen’s model to illustrate how the opinion dynamics arise in the context of social networks.

Let $W=[w_{ij}]$ is a $N\times N$ matrix of interpersonal influence, i.e., for each *i*, $w_{ij}$ denotes for the individual *j*’s social influence to *i*, after normalization *W* satisfies $\sum _{j}w_{ij}=1$. $A={\text{diag}}(a_{1},a_{2},...,a_{N})$ is a $N\times N$ diagonal matrix of individuals susceptibilities to interpersonal influence on the opinion, and satisfies $a_{i}=1-w_{ii}$. In a group of *N* persons, with the initial $N\times 1$ opinions vector $y^{(1)}$, the updating opinions vector $y^{(t)}$ in the interpersonal opinions influence system is described by Eq. (1),

1 $$\begin{aligned} y^{(t+1)}=AWy^{(t)}+(I-A)y^{(1)} \end{aligned}$$

### **Definition 1**

The system (1) reaches the convergence state if, for any initial opinions vector $y^{(1)}$, it holds that $\lim \nolimits _{t\rightarrow \infty }y^{(t)}=y^{*}$.

### **Definition 2**

The system (1) reaches consensus state if, for any initial opinions vector $y^{(1)}$, and each $1\le i,j \le N$, it holds that $\lim \nolimits _{t\rightarrow \infty }|y^{(t)}_{i}-y^{(t)}_{j}|=0$, where |.| is the symbol of the absolute value. This means that, as a result of the social influence process, in the limit they have the same belief on the subject.

As a consequence of system (1), the opinion profile at time $t\in Z\ge 0$ is equal to

2 $$\begin{aligned} y^{(t+1)}=\widehat{W}^{t}y^{(1)}, \end{aligned}$$

where $\widehat{W}^{t}={(AW)^{t}+(\Sigma _{{\text{k}}=0}^{t-1}(AW)^{k})(I-A)}$ is the reduced-form coefficients matrix, discribing the total or net interpersonal effects that transform the initial opinions into equilibrium opinions, and for any entry $\widehat{w}^{t}_{ij}$ in $\widehat{W}^{t}$, satisfies $0\le \widehat{w}^{t}_{ij} \le 1$, $\sum _{j}\widehat{w}^{t}_{ij}=1$. According to Definition 1, under suitable conditions, when $t\rightarrow +\infty$ if $I-AW$ is nonsingular, the system (1) arrives at convergence equilibrium opinions profile $y^{*}$, where $y^{*}=\lim \nolimits _{t\rightarrow \infty }y(t)=(I-AW)^{-1}(I-A)y^{(1)}.$ When $t\rightarrow + \infty$, we have

3 $$\begin{aligned} \lim \limits _{t\rightarrow \infty }\widehat{W}^{t}=\lim \limits _{t\rightarrow \infty }\left\{(AW)^{t}+\sum _{{\text{k}}=0}^{t-1}(AW)^{{\text{k}}}(I-A)\right\}=(I-AW)^{-1}(I-A)=V. \end{aligned}$$

Given large enough time stamp *t*, and a sufficiently small positive real number $\varepsilon$, *V* can be approximated by $\widehat{W}^{t}$. Furthermore, according to the approximation error $||\widehat{W}^{t}-V|| \le \varepsilon$ (where ||.|| denotes the matrix norm), we can obtain the time stamp’s upper bound and lower bound as $ln(||V||-\varepsilon )/ln(||\widehat{W}||)\le t \le ln(||V||+\varepsilon )/ln(||\widehat{W}||)$, where $||\widehat{W}||=||AW+I-A||.$

Followed the same lines of the convergence results by Ishii and Tempo [31], and Golub and Jackson [32], by showing the ergodicity property, Frasca et al. proved the convergence result of system (1); [29]. Touri and Nedic studied the ergodicity and consensus problem with a linear discrete-time dynamic model driven by stochastic matrices [33].

It should be noted according to Defintion 1, that equilibrium opinions may settle on the mean of group members’ initial opinions, a compromise opinion that differs from the initial ones, or altered opinions that does not form a consensus. When consensus is formed in system (1), i.e., as $t\rightarrow +\infty$, $\widehat{W}^{t}$ will have the form of a stratification of individual contributions as following,

$$\begin{aligned} \widehat{W}^{t}=\left[ \begin{array}{cccc} \widehat{w}^{t}_{11} &{}\quad \widehat{w}^{t}_{22} &{}\quad ... &{}\quad \widehat{w}^{t}_{NN} \\ \widehat{w}^{t}_{11} &{}\quad \widehat{w}^{t}_{22} &{}\quad ... &{}\quad \widehat{w}^{t}_{NN} \\ \vdots &{}\quad \vdots &{}\quad \vdots &{}\quad \vdots \\ \widehat{w}^{t}_{11} &{}\quad \widehat{w}^{t}_{22} &{}\quad ... &{}\quad \widehat{w}^{t}_{NN} \end{array}\right] , \end{aligned}$$

which suggests that the initial opinion of each individual makes a particular relative contribution to the emergent consensus.

## Random walk on weighted graph

In this section, without the lose of the generality of system (1), we first introduce the weighted adjacency random matrix, the weighted Laplacian and the transition matrix of the random walk, then we present the conditions for a group opinions consensus under the framework of social influence network model. Here we use the canonical graph symbol *G*(*V*, *E*) in which *V* and *E* denote vertexes and edges respectively.

A weighted directed graph *G* is defined as $w: V\times V \longrightarrow R$ such that $w_{ij}\ne 0$, if $\{i,j\}\not \in E(G)$ then $w_{ij}=0$. In the context, the weighted degree $d_{i}$ of a vertex *i* is defined as $d_{i}=\sum _{j}w_{ij}$, ${\text{vol}}(G)=\sum _{i}d_{i}$ denotes the volume of the graph *G*. For a general weighted directed graph *G*, the corresponding random walk is determined by transition probabilities $p_{ij}=Pr(x_{t+1}=j|x_{t}=i)=w_{ij}/d_{i}$, which are independent of *i*. Clearly, for each vertex *i* satisfies $0\le p_{ij}\le 1, \sum _{i}p_{ij}=1$, in other words, transition matrix *P* is row stochastic matrix. In addition if for any $j \in V(G)$ satisfying $\sum _{j}p_{ij}=1$, then transition matrix *P* is named double stochastic matrix.

For any fixed time step *t*, we define transition matrix *P* on graph $\widehat{W}^{t}$ without normalization, with entries $p_{ij}=Pr(x_{t+1}=j|x_{t}=i)=\widehat{w}^{t}_{ij}/\widehat{d}^{t}_{i}$, where $\widehat{d}^{t}_{i}=\sum _{j}\widehat{w}^{t}_{ij}$, and matrix *L* as follows:

4 $$\begin{aligned} L_{ij}={\left\{ \begin{array}{ll} \widehat{d}^{t}_i-\widehat{w}^{t}_{ii}&{}\quad \,\, \text {if}\,\, i=j, \\ -\widehat{w}^{t}_{ij} &{}\quad \,\,\text {if} \,\, i \,\, \text { and} \, \, j \text { are adjacent}, \\ 0 &{}\quad \text { otherwise}. \end{array}\right. } \end{aligned}$$

where $\widehat{w}^{t}_{ij}\in \widehat{W}^{t}$ is defined in Equations (2) and (3). Let *T* denote the diagonal matrix with the (*i*, *i*)-th entry having value $\widehat{d}^{t}_{i}$ as following

5 $$\begin{aligned} T=\left[ \begin{array}{cccc} \widehat{d}^{t}_{1} &{}\quad ... &{}\quad ... &{}\quad 0 \\ 0 &{}\quad \widehat{d}^{t}_{2} &{}\quad ... &{}\quad 0 \\ \vdots &{}\quad \vdots &{}\quad \vdots &{}\quad \vdots \\ 0 &{}\quad ... &{}\quad ... &{}\quad \widehat{d}^{t}_{N} \end{array} \right] , \end{aligned}$$

we set $T^{-1}(i,i)=0$ for $\widehat{d}^{t}_{i}=0$, and if $\widehat{d}^{t}_{i}=0$ we say *i* is an isolated vertex. Then the graph $\widehat{W}^{t}$’s Laplacian matrix $\zeta$ is defined to be the form $\zeta =T^{-1/2}LT^{-1/2}$, and each entry in $\zeta$ is listed as following,

6 $$\begin{aligned} \zeta _{ij}= {\left\{ \begin{array}{ll} 1-\frac{\widehat{w}^{t}_{ii}}{\widehat{d}^{t}_i}&{}\quad \,\,\text {if}\,\, i=j\,\, \text {and} \,\, \widehat{d}^{t}_i\ne 0, \\ -\frac{\widehat{w}^{t}_{ij}}{\sqrt{\widehat{d}^{t}_i \widehat{d}^{t}_j}}&{}\quad \,\,\text {if} \,\, i\,\, \text { and}\,\, j \,\, \text { are adjacent}, \\ 0 &{}\quad \,\,\text {otherwise}. \end{array}\right. } \end{aligned}$$

Obviously, $\zeta$ is real number matrix, assume its eigenvalues are all real and non-negative. Let the eigenvalues of $\zeta$ be $\{\lambda _{i}|i=0:N-1\}$ in increasing order of $\lambda _{i}$, such that $0=\lambda _{0}\le \lambda _{1}\le ... \le \lambda _{N-1}$. Then transition matrix *P* satisfies $P=T^{-1/2}(I-\zeta )T^{1/2}$, and $\mathbf 1 TP=\mathbf 1 T$, where **1** is unit vector.

### **Definition 3**

The random walk $P^{m}$ is said to be irreducibility if for any $i,j\in V$, there exists some *t* such that $p^{m}_{ij}>0$. Definition 3 ensures the graph $P^{m}$ is strongly connected.

### **Definition 4**

The random walk $P^{m}$ is aperiodic if the greatest common divisor of the lengths of its simple cycles is 1, i.e., ${\text{gcd}}\;\{m: p^{m}_{ii} > 0\}=1$ for any state *i*.

### **Definition 5**

The random matrix *P* is said to be ergodic if there is an unique $n\times 1$ stationary distribution vector $\pi$ satisfying $\lim \nolimits _{m\rightarrow \infty }P^{m}(y^{(1)})^{'}=\pi ,$ where $'$ is the transpose operation.

### **Definition 6**

The random matrix *P* is convergent if $\lim \nolimits _{m\rightarrow \infty }P^{m}(y^{(1)})^{'}$ exists, for any initial vectors beliefs $y^{(1)}$.

The social influence exchange among the *N* agents may be represented by a graph $G(V,E_{m})$ with the set $E_{m}$ of edges given by $E_{m}=\{(i,j)|p_{ij}^{m}>0\}$. But this condition is not sufficient to guarantee consensus of dynamic system (1) as stated in Ref. [24]. This motivates the following stronger version Definition 7, as addressed in Refs. [35, 36].

### **Definition 7**

(Bounded interconnectivity times). There is some $B\ge 1$ such that for each nodes pairs $(i,j)\in E_{\infty }$, agent *j* sends his/her social impact to neighbor *i* at least once at every *B* consecutive time slots, i.e., the graph $(G(P), E_{m} \bigcup ...\bigcup E_{(m+B-1)})$ is strongly connected. This condition is equivalent to the requirement that there exists $B\ge 1$ such that $(i,j)\in E_{m}\bigcup ...\bigcup E_{m+B-1}$ for all $(i,j)\in E_{\infty }$ and $m\ge 0$.

Definition 5 is the well-known result that aperiodicity is necessary and sufficient for convergence in the case where *P* is strongly connected. In other words, the necessary conditions for the ergodicity of *P* are (i) *irreducibility*, (ii) *aperiodicity*, i.e., Definition 5 is equivalent to Definitions 3 and 4. If Definition 5 holds, Definition 6 is satisfied.

If a Markov chain is irreducible and aperiodic, i.e., Definition 3 (or Definition 3’s stronger version Definition 7) and Definition 4 are both satisfied, or equivalently Definition 5 holds, then *P* converges to its corresponding steady distribution. This conclusion is fairly easily verified by adapting theorems on steady-state distributions of Markov chains, such as the proof provided in Ref. [37]. From another alternative, we will prove this result by spectrum graph theorem in the following section.

For above Definitions 3–7, we summarize the associated results in the following Theorem 1, then we emphasize on consensus result proof.

### **Theorem 1**

If *P* is a random matrix, the following are equivalent:

(i) *P* is aperiodic and irreducible.
(ii) *P* is ergodic.
(iii) *P* is convergent, there is a unique left eigenvector $p_{s}$ of *P* corresponding to eigenvalue 1 whose entries sum to 1 such that, for every $y^{(1)}$, $(\lim \nolimits _{m\rightarrow \infty }P^{m}(y^{(1)})^{'})_{i}=\pi (i), where\quad \pi (i)=(p_{s})^{'}(y^{(1)})^{'}$ for every *i*.

Both (i) and (ii) in Theorem 1 are the well-known results. Next we focus on the proof of (iii) based on spectral graph theory. Theorem 1 presents the conditions for the formation of opinions convergence.

## The convergence of opinions profile on random graph

In this section, with the above Definitions 3, 4 or 7, we prove that the convergence of group opinions over general weighted and undirected random graph are almost surely. In addition, we prove the lower bounds on the convergence time *t* for random walk $P^{t}$ to be close to its stationary distribution, given an arbitrary initial distribution and small positive error $\epsilon$. We note that this proof is based on spectrum graph theorem, which is different with Markov chains methods, such as in [20–22, 29].

*Proof* In a random walk associated with a weighted connected graph *G*, the transition matrix *P* satisfies $\mathbf 1 TP=\mathbf 1 T,$ where **1** is the vector with all elements are scalar 1. Therefore, the stationary distribution is exactly $\pi =\mathbf 1 T/{\text{vol}}(G)$. We show that for any initial opinions profile distribution $y^{(1)}$, when *m* is large enough, $P^{m}y^{(1)}$ converges to the stationary distribution $\pi$ in the sense of $L_{2}$ or Euclidean norm. We write $y^{(1)}T^{-1/2}=\sum _{i}a_{i}e_{i}$, where $e_{i}$ denotes the orthonormal eigenfunction associated with $\lambda _{i}$. Because $e_{0}=\mathbf 1 T^{1/2}/\sqrt{{\text{vol}}(G)}$ and ${{<}y^{(1)}},\mathbf 1 >=1$, ||.|| represents the $L^{2}$ norm, we have $a_{0}=\frac{<y^{(1)}T^{-1/2},\mathbf 1 T^{1/2}>}{||1T^{1/2}||} =\frac{1}{\sqrt{vol(G)}}$. We then have

7 $$\begin{aligned}&||y^{(1)}P^{m}-\pi ||=||y^{(1)}P^{m}-\mathbf 1 T/{\text{vol}}(G)|| =||y^{(1)}P^{m}-a_{0}e_{0}T^{1/2}||\nonumber \\&=||y^{(1)}T^{-1/2}(I-\zeta )^{m}T^{1/2}-a_{0}e_{0}T^{1/2}||=|| \sum _{i\ne 0}(1-\lambda _{i})^{m} a_{i}e_{i}T^{1/2}||\nonumber \\&\quad \le (1-\lambda ^{'})^{m}\frac{{\text{max}}_{j}\sqrt{\widehat{d}^{t}_{j}}}{{\text{min}}_{j} \sqrt{\widehat{d}^{t}_{j}}}\le e^{-m\lambda ^{'}}\frac{{\text{max}}_{j}\sqrt{ \widehat{d}^{t}_{j}}}{{\text{min}}_{j} \sqrt{\widehat{d}^{t}_{j}}} \end{aligned}$$

where

$$\begin{aligned} \lambda ^{'}= {\left\{ \begin{array}{ll} \lambda _{1},&{} \text {if}\ 1-\lambda _{1}\ge \lambda _{N-1}-1 \\ 2-\lambda _{N-1},&{} \text {else}. \\ \end{array}\right. } \end{aligned}$$

Given any $\epsilon >0$, for Eq. (7) we have

8 $$\begin{aligned} e^{-m\lambda ^{'}}\frac{{\text{max}}_{j}\sqrt{\widehat{d}^{t}_{j}}}{{\text{min}}_{j}\sqrt{ \widehat{d}^{t}_{j}}}\le \epsilon , \end{aligned}$$

then we have $\frac{{\text{max}}_{j}\sqrt{\widehat{d}^{t}_{j}}}{\epsilon {\text{min}} _{j}\sqrt{\widehat{d}^{t}_{j}}}\le e^{m\lambda ^{'}},$ so $m\ge \frac{1}{\lambda ^{'}}log\left(\frac{{\text{max}}_{j}\sqrt{\widehat{d}^{t}_{j}}}{\epsilon {\text{min}_{j}\sqrt{\widehat{d}^{t}_{j}}}}\right).$

With the symmetry of transition probability $P^{m},$ we easily check that $||y^{(1)}P^{m}-\pi ^{'}||=||(y^{(1)}P^{m}-\pi ^{'})^{'}||=||(y^{(1)} P^{m})^{'}-\pi ||=||(P^{m})^{'}(y^{(1)})^{'}-\pi ||=||P^{m}(y^{(1)})^{'}-\pi ||.$

With this we conclude that after $m\ge [\frac{1}{\lambda ^{'}}log(\frac{{\text{max}}_{j}\sqrt{\widehat{d}^{t}_{j}}}{\epsilon {\text{min}}_{j}\sqrt{\widehat{d}^{t}_{j}}})]$ steps, the $L_{2}$ distance between $P^{m}(y^{(1)})^{'}$ and its stationary distribution $\pi ^{'}$ is at most $\epsilon$. Thus, $P^{m}$ converges to a matrix with all of whose rows are equal to the positive vector $\pi ^{'}=(\pi _{1},\pi _{2},...,\pi _{N})^{'}$, when a consensus is formed in Friedkin and Johnsen’s model. Accordingly, we have $(\lim \nolimits _{m\rightarrow \infty }y^{(m)})_{i}=\sum _{i=1}^{N}\pi _{i} y_{i}^{(1)}$ almost surely with $\varepsilon$ approximating error corresponding to *t* updating steps.

In the herding example, there is consensus (of sorts), while which could lead to the wrong outcome or misunderstandings (misdirections) for the whole social group, such the “Mob phenomenon” of French revolution described by *Gustave* *LeBon*. In this case, group consensus is equivalent to the unwisdom of crowds. If group consensus to be emerged at certain slot $m^{*}$, such that $y^{(m^{*})}=\frac{1}{N}\sum _{i=1}^{N}y^{(1)}_{j}$, for each *j* in a social group, we say that the society is wise, i.e., each individual arrives the group average initial opinions profile.

One special case of the above theorem is when *P* is a double random matrix. With this condition, the matrix has vector **1** as their common left eigenvector at all times, and, therefore, all the entries of the state vector converge to $(1/N)(\mathbf 1 ^{T} y^{(1)}) \mathbf 1 =(1/N)\sum _{j=1}^{N}y_{j}^{(1)}{} \mathbf 1$, in other words, the mean of the initial *N* individual’s opinion profile, with probability 1. This special case is addressed in Ref. [38], we say this group is a wise social group, as introduced in Ref. [32].

## Numeric simulation

**Fig. 1:**
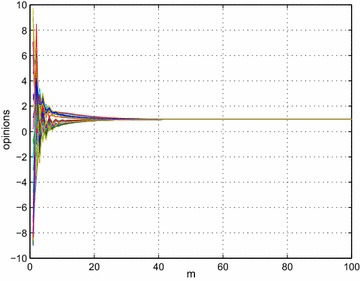
Group opinions dynamics

In this section, we aim to test the theoretical conclusion by numeric simulation methods. We consider the a group (with 34 individuals) discussion processes, and assume that (a) each member is presented with an issue on which opinions could range from −10 to 10 uniformly, (b) independently form an initial opinion on the issue. We fix time $t=1,$ and generate random initial interpersonal influence matrix *W*, with entries between 0 and 7. After realization of *W*, we calculate $max_{j}(\widehat{d}^{t}_{j})=48,$ and $min_{j}(\widehat{d}^{t}_{j})=3.$ Then following the same theoretical line, we construct $P^{t}$ for $t=1,$ compute (4–6) and have $\{\lambda _{i}|i=0:N-1\}.$ According to inequality (7), based on $\{\lambda _{i}|i=0:N-1\}$ we have $\lambda ^{'}= 0.1101$. Given $\epsilon =0.01$, according to (8) we have $m\ge \frac{1}{\lambda ^{'}}log(\frac{{\text{max}}_{j}\sqrt{\widehat{d}^{t}_{j}}}{\epsilon {\text{min}}_{j}\sqrt{\widehat{d}^{t}_{j}}})=54.4184$. The result means that after $m>54$ rounds of discussion and negotiation, the group reaches consensus steady state with preestablished error $\epsilon =0.01$. Figure 1 illustrates the group opinions dynamic processes, we can see that after $m>50$ the difference among members approximate to zero.

Next, we continue simulate Friedkin and Johnsen’s model (1) based on the following assumptions:

1. Prototype group: where group numbers do not know each other, so each one put equal weight on other individuals;
2. Group in evolution: where group numbers have already known each other, so each one might put unequal weight on different individuals according to his/her prior judgements.

Under these two assumptions, we try to find the connection between the behavior of agents and how long the group reaches consensus. Here, we refer to the individuals behaviors as individuals susceptibilities to interpersonal influence on the opinions, or individuals is open minded to take others opinions into account. It is obviously that the diagonals of matrix *A* represent the *susceptible level *(SL) as an parameter measure to describe individuals’ open minded level. Since *I*–*A* is the diagonal of *W*, i.e., ${\text{SL}}= a_{i}=1-w_{ii},i=1, \ldots,N,$ where ${\text{SL}}=0$ means that an agent only looks at his opinion (stubborn or egoistic behavior) and ${\text{SL}}=1$ means that he does not look at his opinion at all, but takes all other opinions into account (open minded or altruistic behavior).

**Fig. 2:**
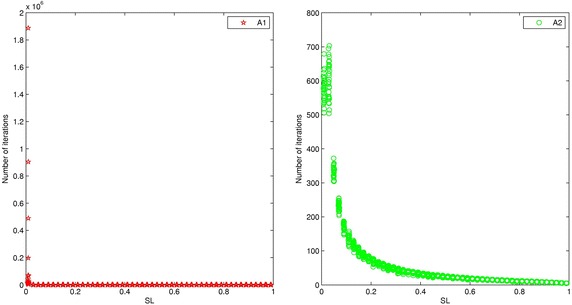
Connection between SL and number of iterations

Under assumption A1, since an agent does not know all the other, that is why he equally takes all other opinion into account. In our simulation, we set equal influence weight for each individual (however with small weights if the group size is larger). Under A2, in order to describe each individual might exert different effects on other individuals, we randomly assign $w_{ij} \in [0,1],i\ne j$. Figure 2 show the connection between *susceptible level *(SL) and how long it takes to reach consensus, within *Prototype group*/*Group in evolution*.

The Fig. 2 also illustrates, under both assumption A1 and A2, if ${\text{SL}}=0$, according to (1) which means that every agent sticks on his opinion, completely without taking care of any other opinion, infinite iterations are needed to reach consensus, means that no consensus will be reached. For a group of very stubborn agent (SL close to 0, but ${\text{SL}} \ne 0$ which means that every agent sticks on his opinion taking less care of any other opinion), the group needs more time to reach consensus. However, carefully examine we observe the dominant difference between A1 and A2: (1) when ${\text{SL}}=0$, both group under assumptions A1 and A2 need enough time to reach consensus, but group A2 needs less convergence time than that of group A1. (2) for *Prototype group* if SL just a little greater than zero, then the group could rapidly toward consensus. However, for a *group in evolution*, because each individual imposes different influence weights on others (this means the group is heterogeneous comparing with *Prototype group*), with the continuously increasing of SL, the number of iterations needed to reach consensus (group consensus time) shows smoothing decaying characteristic.

## Conclusions

In this study, from random walk aspects, we investigate the well-known Friedkin and Johnsen’s model. We define a weighted random walk *P* based on the social influence matrix. If *P* satisfies ergodicity, i.e., aperiodic and irreducible, Friedkin and Johnsen’s model converges to the stable consensus. Furthermore, we prove the lower bounds on the convergence time *m* for random walk $P^{m}$ to be close to its consensus state, given an arbitrary initial opinions profiles and a small achieved convergence tolerance $\epsilon$. We also verify the theoretical result by numeric simulation. Finally, under both *Prototype group* and *Group in evolution* assumptions, we simulate how long the group takes to reach a steady consensus state. We find that with the increasing of *susceptible level *(SL) both *Prototype group* and *Group in evolution* demonstrate opinions convergent characteristics, however, *Prototype group* rapidly tends to consensus if *susceptible level *(SL) bigger than 0.

We hope this study succeeds in providing a rigorous framework to explain and understand group consensus phenomenon. The next work will further consider influence of opinion leaders on population differentiation and the role of the convergence and the control of polarization in Internet group opinions. In addition, because the topology of networks could also be a key factor when opinions spreads among individuals, the networks model in this study may be replaced by small-world, scale free, regular networks, or interdependent network.

Since this paper mainly focuses on the group opinions dynamics over the networked social influence structure, that might ignore the case as social influence mostly follows either independent cascade or linear threshold model. We will combine the cascade or threshold effect into the social influence network model in our future study.

## Footnotes

1 In classical sociological field, social control refers to the occurrence and effectiveness of ongoing efforts in a group to formulate, agree upon and implement collective courses of action.
